# Characterization of medication advertisements in a popular US parenting magazine

**DOI:** 10.15171/hpp.2017.36

**Published:** 2017-09-26

**Authors:** Jennifer Mongiovi, Valerie Cadorett, Corey Basch

**Affiliations:** ^1^Department of Epidemiology, Mailman School of Public Health, Columbia University, NY 10032, USA; ^2^Department of Public Health, William Patterson University, Wayne, NJ 07470, USA

**Keywords:** Advertising, Magazines, Nonprescription drugs, Parenting, Prescription drugs

## Abstract

**Background:** Medication advertisements in magazines typically provide minimal educational benefit. This is of particular concern when targeted to caregivers responsible for making major medical decisions for their children.

** Methods:** A cross-section of 72 issues from Parents magazine were collected and categorized by health condition and availability of the medication by prescription or over-the-counter (OTC).The type of medicine, dose, warning label, indication for child or adult, presence of a cartoon character, and the marketing theme used were documented. Chi-square analysis was used to determine significant differences in content.

** Results:** Fewer than 30% (95% CI: 25.4%, 34.5%) of advertisements contained dosage information and approximately 50% (95% CI: 50.3%, 60.2%) contained side effect warnings. The greatest number of advertisements was for cold, cough and flu medications (14.7%; 95%CI: 11.6%, 18.6%).

**Conclusion:** Medicine advertisements often do not include important information that could help consumers make informed decisions and avoid negative implications. Further research is needed to determine the attitudes of consumers to better understand and support consumers 'needs.

## Introduction

There are many sources of health information, such as TV, radio, magazines, and Internet, which may not present complete and unbiased information. Specifically, direct to consumer (DTC) pharmaceutical advertisements are criticized for their tendency to reduce the influence of medical professionals, and weaken the relationships between physicians and their patients.^1^ The profit motives can affect the advertisements’ content and the minimal educational benefit provided within the advertisement is usually poor.^2^ Consumers need to use caution when exposed to these types of advertisements, which now may include both prescription and over-the-counter (OTC) medications.

In the 1980s, the pharmaceutical industry implemented DTC marketing,^2^ a strategy used by manufacturers to promote their products straight to consumer audiences.^1^ In 1989, $13.1 million was spent on DTC advertising of pharmaceuticals and exceeded $900 million in 1997, more than double than what was spent on advertisements in medical journals.^2^ Consequently, consumers spent over $234 billion on prescription drugs in 2008, doubling from 1999 in the United States.^3^ The use of brand prescriptions drugs were reduced by about 16% in 2014, however, there was a fourfold increase on spending for these prescription medications.^4^ The same Health Care Cost and Utilization Report observed spending on prescription medication increased by $45 per capita between 2013 and 2014, the largest increase in past years for spending on brand prescriptions.

Society tends to believe that prescription medications are not as safe as OTC medications.^5-7^ Although OTC products are often considered less harmful than prescription medications, the Federal Trade Commission has a set of standards that advertisements for these products must abide by.^8^ Cough and cold OTC medications containing dextromethorphan are the most frequently abused.^9^ According to the National Institute on Drug Abuse, there were approximately 4.6 million emergency department visits as a result of drugs in 2009 and 45% of the visits are attributed to drug abuse. A total of 19.1% of the total drug-related emergency visits in 2009 were made by individuals 20 years old or less.^10^ There is a paucity of research that examines marketing tactics used for medicine in general.

Of particular concern is that children are also affected by the influence of medication advertisements. Caregivers are in an important position to make decisions about the use of prescription medicine in children. Between 2007 and 2008, 20% of children reported using at least one prescription drug in last month.^3^ Gu et al identified the most commonly used prescription medication among children less than the age of 6 as the penicillin antibiotic and medication for asthma among children under 11. Central nervous system stimulants were the most commonly used prescription medication used by adolescents (aged 12 to 19) and antidepressants were most commonly used among middle-aged adults (aged 20 to 59).^3^ Many of the advertisements for these prescription medications are targeted not to the immediate consumer, but to the parents responsible for making health decisions for their children. Females have been found to be more likely to have spouses and children that they make medical decisions for, making them the prime target by manufacturers of pharmaceutical and OTC drugs.^11^ In the last year, 61% of Parents’ magazine readers made a direct purchase, spending more than 3 billion dollars on direct purchases.^12^ We therefore identified and described advertisements for medicine in *Parents,* a widely read United States based parenting magazine.

## Materials and Methods

*Parents* had over 13 million readers per month in 2015^12^ with over 11.5 million female readers^13^ with a median reader age of 36 years.^12^ The average age of the females’ children was 7.5 years old.^13^ The median household income of female *Parents*’ readers was $57 179 with a median home value of $202 673.^13^

The cross-section of magazines upon which this study is based consisted of 72 issues from *Parents* magazine from January 2010 to December 2015. The sampling frame consisted of all printed issues over this period. A coding sheet was designed based off of prior studies examining DTC advertisements in this magazine.^14^ Recording the page count for each magazine was the first step of the coding process. Front and inside covers were not included in the page count and tear out promotions were excluded. Advertisements related to weight loss, dietary supplements, and vitamins were not included in the current study as these products are considered more similar to special foods.^15^

Advertisements were categorized by health condition and whether the medication was available by prescription or OTC. Health conditions addressed in advertisements included attention deficit disorder, allergy symptoms, birth control, chronic disease management, cold, cough and flu, cosmetic treatment, head lice, migraine headaches, mood and psychotic disorders, severe allergic reactions, vaccines and anti-viral drugs, and other acute conditions. Chronic diseases were defined by the Department of Health and Human Services Office of the Assistant Secretary of Health selected chronic conditions.^16^

For each medicine advertisement, a single coder documented the kind of medicine, information on dose, presence of a warning label, clear indication for child or adult consumer, presence of a cartoon character, and the marketing theme used. We conducted descriptive analyses that included testing for associations between content and possible themes, advertisement details, or product availability (prescription vs. OTC) using chi-square tests for strength of association. Intra-rater reliability was determined by recoding 9 magazines at a later point (two months) after the initial coding. High agreement was achieved (97%). All analyses were performed using IBM SPSS Statistics version 22 (Armonk, NY).

## Results

Of the 387 advertisements reviewed, 38.8% (n = 150; 5% CI: 34.0%, 43.7%) were targeted to children, 10.9% (n = 42; 95% CI: 8.1%, 14.3%) to either children or adults, and the remaining 50.4% (n = 195; 95% CI: 45.4%, 55.3%) were not specified directly. The greatest number of advertisements was for cold, cough, and flu medicine (14.7%; n = 57; 95% CI: 11.6%, 18.6%), followed by vaccine (13.4%; n = 52; 95% CI: 10.4%, 17.2%) and birth control (11.1%; n = 43; 95% CI: 8.4%, 14.6%) advertisements. Among the medications targeted to children, the most common were cold, cough, and flu (26.0%; n = 39; 95% CI: 19.6%, 33.6%), vaccines (22.7%; n=34; 95% CI: 16.7%, 30.0%), antihistamines (20%; n = 30; 95% CI: 14.5%, 27.1%) and pain relief/anti-inflammatory (20%; n = 30; 95% CI: 14.5%, 27.1%)). For medications targeted to either children or adults, the most common were vaccines (42.9%; n = 18; 95% CI: 29.1%, 57.8%) and epinephrine (26.2%; n = 11; 95% CI: 15.3%, 41.1%). Among non-specified advertisements, the most common were for birth control (22.1%; n = 43; 95% CI: 16.8%, 28.4%), ADD/ADHD medication, and migraines and headaches (12.3%; n = 24; 95% CI: 8.4%, 17.7%) (Table 1).

The most commonly used advertisement themes were peace of mind (26.9%; n = 104; 95% CI: 22.7%, 31.5%), happiness (11.9%; n = 56; 95% CI: 11.3%, 18.3%), and relief (11.4%; n = 44; 95% CI: 8.6%, 14.9%). The theme of becoming smarter was used a significantly higher amount in advertisements targeted to children (n = 9, *P *= 0.011) as well as relief (n = 29, *P *< 0.001). The theme of being “cool” was found exclusively in advertisements targeted to either children or adults (n = 18, *P *< 0.001). Happiness (n = 11, *P *= 0.004) and hope (n = 12, *P *< 0.001) were also most commonly used in advertisements for adults or children. Peace of mind (n = 65, *P *< 0.001) and sadness (n = 40, *P *< 0.001) were found predominantly in advertisements not targeted to a specific age range. Among advertisements for children and unspecified audience, the most common theme was peace of mind (26.0%; n = 39; 95% CI: 19.6%, 33.6%; 33.3%; n = 65; 95% CI: 27.1%, 40.2%). Hope was most common among advertisements for children or adults (28.6%; n = 12; 95% CI: 17.2%, 43.6%).

Although only 16.8% (n = 65; 95% CI: 13.4%, 20.9%) of all advertisements contained a cartoon image, these were more likely to be found in advertisements for children’s products (n = 28, *P *< 0.001). Dosage indication was only present in 29.7% (n = 115; 95% CI: 25.4%, 34.5%) of all advertisements and side effect warnings were only present in 55.3% (n = 214; 95% CI: 50.3%, 60.2%). Advertisements targeted to either children or adults were most likely to contain this information (97.6%; n = 41, *P *< 0.001 for both).

## Discussion

Our study found that fewer than 30% of advisements contained dosage information and a little over 50% contained side effect warnings. This is critical for parents to know and be aware of. An important study found that only 30% of the parents studied could demonstrate the ability to correctly measure liquid medication and identify the correct medication dose for their child.^17^ The greatest number of advertisements in our study was for cold, cough, and flu medications (14.7%). These types of medications contain ingredients that are psychoactive when taken at doses higher than recommend and are often abused because of this.^18^ Roughly 1 in 30 teens reported using OCT cough medications to get high.^19^

This study is novel in that it looks specifically at medicine advertisements in a highly popular US based magazine for parents. As women are the main readers of these magazines, it is important to understand the types of medicines featured, as well as the marketing strategies used. According to the Harvard Business Review, women control approximately $20 trillion in yearly consumer spending globally and that figure may climb to new heights over the next few years. Large sums of money are spent by companies to better understand the female market, such as Johnson & Johnson who spends 4% of its sales on consumer research and development.^20^ Wilkes et al found that women were more likely to targeted by drug advertisements than men and the drug advertisements targeted the user not third party intermediaries.^21^ Additionally, the study found that females were more likely to be aware of drug advertisements than males.^21^ DTC ads may serve as a vehicle to convey health information in regards to diagnosis and treatment choices. Women are frequently the primary family caregivers and tend to be asked about treatment and symptom control for their family.^22^

## Conclusion

This study supports the current yet limited evidence that the driving force behind both DTC advertisement and OTC product advertisement may be profit in lieu of information. While electronic forms of media engulf modern consumers, print media continues to play a critical role as a source of health information as well.^22^ There may be greater ability to provide information in print advertisements, but this information is often presented in hardly visible type or excluded altogether by referencing that more information is available online. This study is limited by the observational design, however, has yielded additional hypotheses. Further research is needed to determine the attitudes of consumers towards these advertisements to better understand and support consumers’ needs.

## Ethical approval

As this study did not involve human subjects, it was considered exempt from review by the Institutional Review Board (IRB) at William Paterson University and Teachers College, Columbia University.

## Competing interests

The authors declare that they have no competing interests.

## Funding

This was an unfunded analysis.

## Authors’ contributions

JM conceived the research hypothesis, performed analysis, and assisted in manuscript preparation. VC assisted in the conception of the research hypothesis, collected the data, and assisted in manuscript preparation. CHB designed the study and assisted in manuscript preparation.

**Table 1:** Advertisement characteristics by targeted consumer (n = 387)

		**Targeted consumer**			***P *** **value** ^a^
	**Total **	**Children**	**Children or adults**	**Not specified**	
	**No. (%) **	**No. (%) **	**No. (%) **	**No. (%) **	
	**387 **	**150 (38.8) **	**42 (10.9) **	**195 (50.4) **	
**Type of medication**					
ADD/ADHD	24 (6.2)	0 (0.0)	0 (0.0)	24 (12.3)	
Antibody/antiviral	9 (2.3)	0 (0.0)	0 (0.0)	9 (4.6)	
Antihistamine	37 (9.6)	30 (20.0)	1 (2.4)	6 (3.1)	
Birth control	43 (11.1)	0 (0.0)	0 (0.0)	43 (22.1)	
Chronic disease management^a^	15 (3.9)	0 (0.0)	0 (0.0)	15 (7.7)	
Cold, cough, and flu	57 (14.7)	39 (26.0)	0 (0.0)	18 (9.2)	
Epinephrine	27 (7.0)	0 (0.0)	11 (26.2)	16 (8.2)	
Gastrointestinal agents	14 (3.6)	2 (1.3)	0 (0.0)	12 (6.2)	
Head lice treatment	17 (4.4)	0 (0.0)	5 (11.9)	12 (6.2)	
Migraine and headache	24 (6.2)	0 (0.0)	0 (0.0)	24 (12.3)	
Mood and psychotic disorders	20 (5.2)	0 (0.0)	0 (0.0)	20 (10.3)	
Oral care	6 (1.6)	6 (4.0)	0 (0.0)	0 (0.0)	
Pain relief/ anti-inflammatory	30 (7.8)	30 (20.0)	0 (0.0)	0 (0.0)	
Sleep aid	3 (0.8)	0 (0.0)	0 (0.0)	3 (1.6)	
Supplement	9 (2.3)	9 (6.0)	0 (0.0)	0 (0.0)	
Vaccine	52 (13.4)	34 (22.7)	18 (42.9)	0 (0.0)	
**Advertisement theme** ^c^					
Adventure	9 (2.3)	6 (4.0)	0 (0.0)	3 (1.5)	0.184
Become smarter	11 (2.8)	9 (6.0)	0 (0.0)	2 (1.0)	0.011
Being “cool”	18 (4.7)	0 (0.0)	18 (42.9)	0 (0.0)	<0.001
Fear	3 (0.8)	0 (0.0)	0 (0.0)	3 (1.5)	0.226
Happiness	46 (11.9)	19 (12.7)	11 (26.2)	16 (8.2)	0.004
Hope	30 (7.8)	0 (0.0)	12 (28.6)	18 (9.2)	<0.001
Peace of mind	104 (26.9)	39 (26.0)	0 (0.0)	65 (33.3)	<0.001
Prevention	25 (6.5)	10 (6.7)	1 (2.4)	14 (7.2)	0.513
Relief	44 (11.4)	29 (19.3)	0 (0.00	15 (7.7)	<0.001
Sadness	40 (10.3)	0 (0.0)	0 (0.0)	40 (20.5)	<0.001
**Advertisement characteristics** ^b^					
Contains cartoon image	65 (16.8)	42 (28.0)	0 (0.0)	23 (11.8)	<0.001
Dosage indicated	115 (29.7)	34 (22.7)	41 (97.6)	40 (20.5)	<0.001
Side effect warning	214 (55.3)	34 (22.7)	41 (97.6)	139 (71.3)	<0.001

^a^ Chi-square test for strength of association (α = 0.05). ^b^ Excludes chronic psychiatric conditions. ^c^ Groups not mutually exclusive.
